# Is Satisfaction with the Acute-Care Experience Higher amongst Consumers Treated in the Private Sector? A Survey of Public and Private Sector Arthroplasty Recipients

**DOI:** 10.1371/journal.pone.0159799

**Published:** 2016-08-04

**Authors:** Justine M. Naylor, Joseph Descallar, Mechteld Grootemaat, Helen Badge, Ian A. Harris, Grahame Simpson, Deanne Jenkin

**Affiliations:** 1 Orthopaedic Department, Liverpool Hospital, Liverpool, NSW, Australia; 2 South West Clinical School UNSW, Randwick, NSW, Australia; 3 Ingham Institute of Applied Medical Health Research, Liverpool, NSW, Australia; 4 University of Amsterdam, Amsterdam, Netherlands; 5 Brain Injury Rehabilitation Unit, Liverpool Hospital, Liverpool, NSW, Australia; 6 School of Human Services and Social Work, Griffith University, Gold Coast, Queensland, Australia; 7 John Walsh Centre for Rehabilitation Research, University of Sydney, Sydney, NSW, Australia; Wuhan University, CHINA

## Abstract

**Background:**

Consumer satisfaction with the acute-care experience could reasonably be expected to be higher amongst those treated in the private sector compared to those treated in the public sector given the former relies on high-level satisfaction of its consumers and their subsequent recommendations to thrive. The primary aims of this study were to determine, in a knee or hip arthroplasty cohort, if surgery in the private sector predicts greater overall satisfaction with the acute-care experience and greater likelihood to recommend the same hospital. A secondary aim was to determine whether satisfaction across a range of service domains is also higher in the private sector.

**Methods:**

A telephone survey was conducted 35 days post-surgery. The hospital cohort comprised eight public and seven private high-volume arthroplasty providers. Consumers rated overall satisfaction with care out of 100 and likeliness to recommend their hospital on a 5-point Likert scale. Additional Likert-style questions were asked covering specific service domains. Generalized estimating equation models were used to analyse overall satisfaction (dichotomised as ≥ 90 or < 90) and future recommendations for care (dichotomised as ‘definitely recommend’ or ‘other’), whilst controlling for covariates. The proportions of consumers in each sector reporting the best Likert response for each individual domain were compared using non-parametric tests.

**Results:**

457 survey respondents (n = 210 private) were included. Less patient-reported joint impairment pre-surgery [OR 1.03 (95% CI 1.01–1.05)] and absence of an acute complication (OR 2.13 95% CI 1.41–3.23) significantly predicted higher overall satisfaction. Hip arthroplasty [OR 1.84 (1.1–2.96)] and an absence of an acute complication [OR 2.31 (1.28–4.17] significantly predicted greater likelihood for recommending the hospital. The only care domains where the private out-performed the public sector were hospitality (46.7 vs 35.6%, p <0.01) and frequency of surgeon visitation (76.4 vs 65.8%, p = 0.03).

**Conclusions:**

Arthroplasty consumers treated in the private sector are not more satisfied with their acute-care experience nor are they more likely to recommend their hospital provider. Rather, avoidance of complications in either sector appears to result in improved satisfaction as well as a greater likelihood that patients would recommend their hospital provider.

## Introduction

In many countries, private and public healthcare sectors co-exist to varying degrees. Comparisons in performance between the two sectors are arguably most relevant where both sectors have pronounced roles and essentially compete with each other. In the United States, healthcare is predominantly privately financed whilst in other developed countries such as Canada, the United Kingdom and the Netherlands, most healthcare is provided in the public sector [1]. In Australia, whilst the public system is the larger, the private sector—including commercial, charitable and non-government entities—funds 33% of all healthcare and covers more than half of all surgical episodes performed in operating rooms [2].

Consumer satisfaction with healthcare is one area of performance that is compared and benchmarked [3,4], and for a healthcare landscape such as Australia’s, between-sector comparisons are likely to be of interest to various stakeholders. Consumer satisfaction is arguably a primary goal of private healthcare providers given that repeat business from satisfied customers and their recommendations to others explicitly influence their solvency and, ultimately, their existence [4, 5]. In contrast, though public healthcare providers are required and encouraged by government policy to monitor consumer satisfaction [3], the latter is necessarily secondary to their core goal–the delivery of healthcare within the constraints of pre-determined publically-funded resources [4]. Given the different weights consumer satisfaction must carry in the private and public healthcare sectors, certain aspects of care known to be important to consumer satisfaction–such as the physical environment and information sharing [3]–may also vary across the sectors. Higher levels of satisfaction could reasonably be expected in the private sector if key private sector foci include those aspects of care which influence consumer satisfaction [5]. If consumer satisfaction is not higher in the latter, understanding why this is the case will be important for healthcare providers in a competitive market.

Total knee and hip arthroplasty (TKA, THA) surgeries are commonly performed in Australia as they are elsewhere, with the majority undertaken in the private sector [6]. Whilst satisfaction with the outcome of the surgery has received considerable attention in the peer-reviewed literature [7–13], consumer satisfaction with the acute-care experience has received comparatively little [8,14]. Further, to our knowledge, no previous studies have compared satisfaction of arthroplasty recipients with the acute care experience between those treated in the private and public sectors.

In this study, we hypothesized that arthroplasty recipients treated in the private sector will report higher levels of satisfaction with the acute-care received. The primary aims of this study were to determine if care in the private sector predicts higher overall satisfaction and likeliness of future recommendation for care whilst controlling for factors known to affect satisfaction. Secondarily, we aimed to determine whether satisfaction in specific domains of care is higher amongst arthroplasty recipients treated in the private sector.

## Methods

### Study design and participants

A cross-sectional telephone survey was conducted to capture and compare consumer satisfaction across the two sectors. The study was nested within an ongoing larger, observational study capturing pre-operative, acute-care and longer-term data from osteoarthritis patients undergoing primary TKA or THA (ClinicalTrials.gov NCT01899443). The larger study involved a part-random, part-convenience sample of 19 high-volume sites (defined as performing over 275 knee or hip arthroplasties annually) from five Australian States. All participants provided written, informed consent. The satisfaction survey was approved as a sub-study within the larger study by several ethics committees: Hunter New England HREC (NSW); St Vincent's Health and Aged Care HREC (Queensland); Austin Health HREC (Victoria); Barwon Health HREC (Victoria); Epworth HREC (Victoria); Calvary Health Care Clinical and Research Ethics Committee (Tasmania) and; Calvary Healthcare Adelaide HREC (South Australia)—and carried out under the principals expressed in the Declaration of Helsinki. Some hospitals were excluded (n = 2) due to expected delays in obtaining ethical approval for the sub-study. The satisfaction data were collected from participants in the larger study between February 2014 and February 2015.

### Baseline data and acute-care outcomes

A consecutive series of eligible people who provided informed consent to participate in the observational study provided basic demographic, sociodemographic and comorbid data during a pre-admission visit or telephone call 2–6 weeks prior to surgery. They also completed patient-reported outcomes measures (PROMS). Acute care outcomes such as complications and length of stay were provided by the sites using a standardised pro-forma. Thirty-five day complication and PROM data were obtained via telephone follow-up by trained study personnel not involved in care delivery at any site. Participant ineligibility included revision surgery, documented dementia, rheumatoid arthritis in the joint being replaced and another arthroplasty within three months of the pending surgery.

The Oxford Knee or Hip Score (OKS, OHS) [15] and the EuroQol visual analogue scale for ‘health today’(EQ VAS) [16] were used to capture PROM data. In brief, the OKS and OHS comprise 12 joint-specific Likert-style questions each concerning pain and functional impairment over the last four weeks. Each item is scored from zero (maximal discomfort/pain or maximal impairment) to four (no pain/discomfort or impairment), providing a total score out of 48 with higher scores indicating better joint status. The EQ VAS asks respondents to rate their health ‘today’ on a continuous scale, zero indicating worse health imaginable and 100 indicating best health imaginable.

For this nested study, complications were coded according to the Clavien-Dindo Classification of Surgical Complications [17] where complications are defined as any event or outcome that necessitates a deviation from normal management and the classification applied is based on the type of deviation (Table 1). Pilot data revealed that both transient and seemingly benign adverse events–such as persistent vomiting or diarrhoea—did influence consumer satisfaction thus a coding system that permitted the capture of a range of problems of varying severity was required. Complications experienced during the acute-care period were used in the analysis.

**Table 1:** Classification of complications.

Grade	Definition	Comments
**I**	Any deviation from the normal postoperative course without the need for pharmacological treatment or surgical, endoscopic and radiological interventions. Acceptable therapeutic regimens: drugs as anti-emetics, antipyretics, analgesics, diuretics and electrolytes and physiotherapy. This grade also includes wound infections opened at the bedside.	Included persistent diarrhoea, nausea or vomiting
**II**	Requiring pharmacological treatment with drugs other than such allowed for grade I complications. Blood transfusions and total parenteral nutrition are also included.	The need for blood transfusion alone was not included as a complication here
**III**	Requiring surgical, endoscopic or radiological intervention	
**IIIa**	Intervention not under general anesthesia	
**IIIb**	Intervention under general anesthesia	
**IV**	Life-threatening complication (including CNS complications) requiring IC/ICU-management	Included blood pressure instability requiring transfer to high-dependency
**IVa**	Single organ dysfunction (including dialysis)	
**IVb**	Multi organ dysfunction	
**V**	Death of a patient	Excluded here as deceased patients unable to be surveyed

Above classifications based on the Clavien-Dindo Classification [17].

### Satisfaction data collection methods

#### Survey development

*Content validity*: Prior to conducting the definitive survey, a series of broad questions providing opportunities for open discourse were piloted with 40 participants. Review of the responses to these questions revealed their broadness failed to discriminate either between people or the differing issues that contributed towards overall satisfaction with the acute-care experience. From the open-ended responses, however, recurrent themes emerged regarding factors that influenced patient perception of the care received. These themes included accessibility and efficiency of staff, staff attitudes, quality of hospital food and hospital environment, communication between staff and patients, and the experience of an adverse event or complication even if minor. From discussions within the research team around the emergent themes, together with reference to the domains well explored in the healthcare consumer satisfaction literature [3,18,19], the survey was refined (S1 Appendix). Seventeen questions were asked covering the following satisfaction domains: physical environment and hospitality; staff communication and information sharing; availability of staff and frequency of care; quality of care; safety; overall satisfaction; and future recommendations for care. One question required respondents to rate overall satisfaction with their healthcare out of 100, anchored by 0 (no satisfaction) to 100 (extremely satisfied). Twelve questions were rated on a Likert-type scale format in which patients could choose from four or five different alternatives, typically ranging from ‘very satisfied’ to ‘very dissatisfied’. To reduce positive response bias, a neutral category ('neither satisfied nor dissatisfied') was included. Two questions required respondents to recall the frequency with which they were treated or visited by their physiotherapist or surgeon in hospital. Finally, two open-ended questions asked respondents to specify what aspect of care they were most satisfied with and what aspect they considered required the most improvement. The open-ended questions were added to provide specific feedback to the sites involved as recommended [18], but are not included in the analysis here. In brief, the open-ended responses generally explained the responses to specific items already asked about in the closed-ended questions. For example, dissatisfaction with nurse responsiveness was often accompanied by a comment about how overworked nursing staff appeared to be.

*Reproducibility*: As part of survey development, a subset of 41 participants (n = 25 TKA; n = 16 THA; 20 females; mean age of 67 yr) repeated the satisfaction survey one week after their 35-day interview. Four interviewers participated in the test-retest component, increasing the generalisability of the findings, but the same interviewer repeated the survey for an individual participant for within-interviewer stability. Percentage agreement and the Kappa or weighted Kappa statistic (or coefficient) [20–23] were used to quantify the week-to-week precision (in this case, repeatability) of the 12 close-ended questions. Interpretation of the Kappa values was according to the following convention [20,24,25]: values < 0.2 reflect poor or slight strength of agreement; values between 0.21 and 0.40, reflect fair agreement; values between 0.41 and 0.6, reflect moderate agreement; values from 0.61 to 0.8 reflect good; and values 0.81 to 1 are very good. Paired t-tests and intra-class correlations (ICC) (one-way random effects) were used to determine the repeatability of the three continuous scale questions. For ICCs, values of 0.7 or more are considered to have acceptable reliability [26].

The tabulated results for the reproducibility testing are provided in S2 Appendix. Eight of the 12 ordinal survey questions obtained a Kappa reflecting ‘good agreement’; three achieved a ‘moderate’ level of agreement; and one achieved a ‘fair’ level of agreement. Raw agreement, expressed as percentage agreement, exceeded 84% for eight of the questions, and the lowest percentage agreement observed was 71% (‘nurse call responsiveness’). The question achieving ‘fair’ agreement was associated with a raw agreement of 90%. Agreement amongst the survey questions requiring a scaled or continuous response was acceptable for ‘surgeon visits’ and ‘overall satisfaction’, but not for ‘physiotherapy visits’. ‘Physiotherapy visits’ as a continuous variable was subsequently excluded from further analysis.

### Analysis of the definitive survey

Descriptive statistics were used to compare the two sectors in terms of their characteristics and unadjusted outcomes. Additionally and a priori, factors known to affect satisfaction, and thus could subsequently be controlled in the analysis, were identified and generalized estimating equation models were used to model the ‘overall satisfaction’ outcome. The latter was dichotomised (<90, ≥ 90) owing to its non-normal distribution. Models were clustered by hospital to account for a cluster effect if present. In terms of variables considered relevant to control, patient expectations for care were not directly captured, but variables considered proxies for expectation (age and education level)[19] were included. Other independent variables included binary, ranked or nominal-type variables–sector, gender, joint (knee or hip), surgery type (unilateral or bilateral), presence of an acute complication, American Society of Anesthesiology (ASA) score–as well as continuous variables such as body mass index (BMI), baseline Oxford score, and EQVAS. All variables (except for sector) were partitioned to distinguish between the individual patient effect and the hospital effect. The same analysis was conducted to determine factors relating to the outcome ‘future recommendation of care’ with likelihood to recommend dichotomised into ‘Definitely’ and ‘Other’.

For the secondary outcomes, comparisons between the two sectors for the individual domains service were made using Chi square (χ^2^) tests or Fisher’s exact test as appropriate. Specifically, the proportions stating the top response for each domain, with all other responses categorised as ‘other’, were compared. The dichotomy (top response vs other) was necessitated due to a large majority of responses always being across the top two responses for both sectors, indicating that, in the main, people were almost always satisfied or highly satisfied. Thus, the most relevant comparison was deemed to be one that compared the proportion stating the best or top response for each domain.

To assess which individual domain items were related to the global response for overall satisfaction–and thus identify if any service domain in particular may be ‘explaining’ overall satisfaction—the entire cohort was stratified based on their overall satisfaction rating (< 90, ≥ 90). The individual domain responses of the two groups were then compared using χ^2^ tests. If the group reporting the highest overall satisfaction also had a higher proportion providing the best response for a given domain item, then that item was considered to be contributing to global overall satisfaction. The comparisons were repeated when the cohort was stratified based on the future recommendation for care responses (‘definitely recommend’ versus all other options).

### Sample size

Assuming a between-group absolute difference of 10% in the proportion of respondents providing the top answer for each primary outcome would represent an important minimum difference in consumer satisfaction, 271 respondents in each group (sector) (542 in total) was required (power 80%, α 0.05), assuming an intra-class correlation of 0.01.

## Results

All consenting consecutive persons over the time the satisfaction survey was conducted participated in the survey. Recruitment necessarily ceased after 509 respondents as the end date for the larger study was met, and two sites did not have any respondents as all their participants had already undergone their 35-day follow-up by the time the sub-study commenced. Of the 509 respondents, 487 had complete data sets. Another 30 respondents who had bilateral surgery were excluded as their inclusion made the models unstable due to their uneven distribution between the private (n = 22) and public sectors (n = 8). Thus, 457 were included in the final analysis. Contributions from each hospital ranged from 12 to 134 respondents. The characteristics of the cohort, categorised by sector and surgery type, are summarised in Table 2. The private sector group differed significantly from the public sector group in terms of the baseline Oxford scores and the education profile.

**Table 2:** Characteristics of the analysed cohort by arthroplasty type and sector.

	Total knee arthroplasty,		Total hip arthroplasty,	
	N = 256		N = 201	
	Private,	Public,	Private,	Public,
	n = 106	n = 150	n = 104	n = 97
**Age, mean (sd)**	68 (8)	67 (9)	65 (10)	66 (11)
**Gender, female, n**	67 (63%)	95 (63%)	50 (48%)	40 (41%)
**Body mass index, mean (sd)**	31 (6)	34 (8)	28 (5)	30 (6)
**ASA, mode**				
**1**	11 (10%)	9 (6%)	21 (20%)	8 (8%)
**2**	57 (54%)	86 (57%)	52 (50%)	60 (62%)
**3**	36 (34%)	55 (37%)	30 (29%)	29 (30%)
**4**	2 (2%)	0 (0%)	1 (1%)	0 (0%)
Education level*				
**0**	0 (0%)	2 (1%)	0 (0%)	0 (0%)
**1**	27 (25%)	77 (51%)	22 (21%)	40 (41%)
**2**	67 (63%)	69 (46%)	58 (56%)	53 (55%)
**3**	12 (11%)	2 (1%)	24 (23%)	4 (4%)
**Comorbidity ≥ 1, n**	95 (92%)	136 (94%)	85 (84%)	81 (86)
**EQ5D-VAS, median**	75 (IQR 65–85)	70* (IQR50-85)	75 (IQR 60–85)	75 (IQR60-80)
**Oxford knee or hip score, mean (sd)**	24 (8)	20 (9)*	24 (9)	18 (8)**
**Complication ≥ 1, n**	39 (37%)	35 (23%)*	17 (16%)	16 (16%)
**Complication according to Clavien-Dindo Index**				
**0**	66 (62%)	113 (75%)	87 (84%)	80 (82%)
**I**	16 (15%)	20 (13%)	9 (9%)	8 (8%)
**II**	18 (17%)	9 (6%)	6 (6%)	6 (6%)
**III**	1 (1%)	1 (1%)	0 (0%)	1 (1%)
**IV**	0	1 (1%)	0 (0%)	1 (1%)
**V**	5 (5%)	6 (4%)	2 (2%)	1 (1%)

ASA, American Society of Anesthesiology; IQR, inter-quartile range.

*p<0.01.

Unadjusted overall satisfaction and future recommendations for care are shown in Table 3. There was a trend for overall satisfaction (median and proportion stating the top answer), prior to adjustment for covariates, to be slightly higher amongst TKA recipients treated in the public sector. Likeliness of recommending the same hospital to others was significantly greater amongst public TKA consumers. There were no significant differences in the unadjusted outcomes for THA respondents. For the combined TKA and THA cohort, univariate analysis which accounted for clustering, demonstrated that public sector consumers were significantly more satisfied than private sector consumers (OR 1.56 95% CI 1.16–2.10, p<0 01), but there was no significant difference (p = 0.85) for likeliness to recommend between the sectors.

**Table 3:** Unadjusted outcomes: overall satisfaction and future recommendations for care.

	TKA			THA		
	Public,	Private,	p-value	Public,	Private,	P-value
	n = 150	n = 106		n = 97	n = 104	
**Satisfaction,**	95	92.5	0.159	95	95	0.14
**median**	(90–100)	(85–99)		(90–100)		
**(IQR)**					(90–100)	
**Satisfaction, ≥ 90**	124 (83%)	77 (73%)	0.06	76 (78%)	79 (76%)	0.74
**Definitely recommend**	135 (90%)	85 (80%)	0.03	88 (91%)	98 (94%)	0.42

Table 4 summarises the multivariate analyses for the predictors of overall satisfaction. ‘Sector’ was not a significant predictor. Less joint-specific impairment at baseline (Oxford Score) (OR 1.03 95% CI 1.01–1.05, p = 0.009) and the absence of a complication (OR 2.13 95% CI 1.41–3.23, p = 0.001), predicted higher overall satisfaction. Despite the significant association between the presence or absence of a complication, the effect was not straight-forward as 66% of respondents with a complication were highly satisfied and 26% of those without a complication were less than highly satisfied.

**Table 4:** Multivariate analyses for the prediction of high-level satisfaction (≥90% or < 90%).

	OR	Lower 95% CI	Upper 95% CI	P-value
**Sector**				
**Public**	1.78	0.65	4.88	0.26
**Private**	1	-	-	-
**Age**	1.00	0.96	1.04	0.95
**Body mass index**	1.02	0.99	1.05	0.20
**Surgery Type**				
**Total knee arthroplasty**	1.09	0.68	1.73	0.74
**Total hip arthroplasty**	1	-	-	-
**Oxford**	1.03	1.01	1.05	**0.01**
**EuroQol VAS**	1.00	0.99	1.08	0.53
**ASA**				
**1,2**	1.29	0.86	2.01	0.27
**3,4**	1	-	-	-
**Complication**				
**No Complication**	2.13	1.41	3.23	**<0.01**
**Complication**	1	-	-	-
**Education**				
**Year 10 and below**	1.16	0.73	1.83	0.53
**Year 11, Year 12, Tertiary**	1	-	-	-

VAS- visual analogue scale; ASA—American Society of Anesthesiology. Model adjusted for between hospital effects of Age, BMI, Surgery Type, Oxford score, EQ5D, ASA, Complication and Education.

Table 5 summarises the multivariate analysis for the predictors of future recommendations for care. Sector was not a significant predictor. Hip surgery (OR 1.84 95% CI 1.14–2.96, p < 0.01) and the absence of a complication (OR 2.31 95% CI 1.28–4.17, p < 0.01] predicted whether the person would definitely recommend their hospital to another person. As for overall satisfaction, the association between a complication and future recommendation was not straight-forward with 79% of those with a complication still ‘definitely likely’ to recommend the hospital to others.

**Table 5:** Multivariate analyses for the prediction of future recommendation of the same hospital to others (‘Definitely recommend’ or ‘Other’).

	OR	Lower 95% CI	Upper 95% CI	P-value
**Sector**				
**Public**	1.63	0.19	14.26	0.66
**Private**	1	-	-	-
**Age**	0.99	0.96	1.01	0.28
**Body mass index**	0.99	0.95	1.02	0.38
**Surgery Type**				
**Total hip arthroplasty**	1.84	1.14	2.96	**0.01**
**Total knee arthroplasty**	1	-	-	-
**Oxford**	1.00	0.98	1.03	0.84
**EuroQol VAS**	1.01	1.00	1.03	0.14
**ASA**				
**1,2**	0.77	0.41	1.43	0.4
**3,4**	1	-	-	-
**Complication**				
**No complication**	2.13	1.28	4.17	**<0.01**
**Complication**	1			
**Education**				
**Year 10 and below**	1.22	0.82	1.81	0.33
**Year 11, Year 12, Tertiary**	1	-	-	-

VAS- visual analogue scale; ASA—American Society of Anesthesiology Model adjusted for between hospital effects of Age, BMI, Surgery Type, Oxford score, EQ5D, ASA, Complication and Education.

As the presence of a complication was significant for both overall satisfaction and future recommendation for care, we performed sensitivity analyses to assess whether pooling both major and more minor complications affected the results. The models remained the same regardless of whether or not we included the more minor complications in the analyses.

Table 6 summarises the satisfaction associated with specific individual domains of care or service by private or public sector. The private sector had a significantly higher proportion reporting the top response for two domains–hospitality (food) and surgeon frequency of visitation. The public sector had a higher proportion reporting the top response for ‘Nurse’, ‘Doctor’ and ‘Anaesthetist communication’. Regarding recalled frequency of surgeon visitation, private respondents reported a significantly higher rate of visitation (number of visits / length of stay) compared with public respondents (0.57 (sd 0.35) vs 0.34 (sd 0.32), p < 0.01).

**Table 6:** Proportion per sector reporting the best (top) response.

	Public,	Private,	P-value
	%	%	
**Cleanliness: very satisfied**	77.3	82.4	0.44
**Food: very satisfied**	35.2	47.6	<0.01
**Nurse communication: very satisfied**	85	76.7	0.02
**Ward doctor communication: very satisfied**	73.7	60.5	0.02
**Anaesthetist communication—anaesthetic options: very clearly**	89.9	81.9	<0.01
**Anaesthetist communication—pain management options: very clearly**	77.1	62.6	<0.01
**Nurse call responsiveness: straight away**	69.6	55.1	0.07
**Surgeon visit frequency: very satisfied**	65	76.9	0.01
**Physiotherapist visit frequency: very satisfied**	63.7	58.2	0.07
**Physiotherapy care: very satisfied**	69.1	61.5	0.17
**Sufficient staff numbers: always enough**	72.5	70.5	0.96

Table 7 summarises the pattern of responses to individual domains according to overall satisfaction grouping or likeliness to recommend grouping. For every service or care domain, the most highly satisfied group had a significantly higher proportion of respondents reporting the top response, and this was true when the cohort was stratified by likeliness to recommend. These group trends suggest that all domains appeared to be contributing to both primary outcomes. Table 7 also indicates, however, that being highly satisfied overall did not guarantee that the respondent was necessarily highly satisfied with all aspects of care. Reference to the Food domain, as an example, shows that a minority (only 48%) of those who were highly satisfied overall, were actually very satisfied with the food.

**Table 7:** Proportion of respondents in the ‘Overall satisfaction’ group (≥90%, <90%) and ‘Likely to recommend’ group (Definitely or Other) reporting the top response in each service domain.

	Overall satisfaction grouping		Likely to recommend grouping	
	≥90%	<90%	Definitely	Other
**Cleanliness: very satisfied**	87%	42%	85%	37%
**Food: very satisfied**	48%	16%	45%	12%
**Nurse communication: very satisfied**	90%	50%	86%	41%
**Ward doctor communication: very satisfied**	75%	41%	72%	35%
**Anaesthetist communication—anaesthetic options: very clearly**	90%	72%	88%	69%
**Anaesthetist communication—pain management options: very clearly**	75%	54%	73%	44%
**Nurse call responsiveness: straight away**	69%	44%	67%	29%
**Surgeon visit frequency: very satisfied**	77%	49%	75%	32%
**Physiotherapist visit frequency: very satisfied**	70%	30%	66%	20%
**Physiotherapy care: very satisfied**	74%	35%	70%	31%
**Sufficient staff numbers: always enough**	76%	54%	73%	59%

Note: All between-group comparisons were significantly different at the p < 0.001 level.

## Discussion

We hypothesised that arthroplasty consumers treated in the private sector may be more satisfied with their acute care experience compared to their public counterparts. Our study indicates that whilst private sector care recipients do report higher satisfaction for some care domains, they do not, in the main, report higher overall satisfaction and are no more likely than public sector consumers to recommend their hospital provider to others. In general, we observed, as have others [18,19], that global satisfaction ratings are high despite instances where the care was perceived to be less than very satisfactory in specific domains. It has been suggested, therefore, that when overall satisfaction scores tend to be high, reference to individual domain items may provide more useful feedback to the care provider [18].

The limited literature available is inconsistent with respect to whether the type of facility (public or private) or whether insurance status predicts satisfaction with healthcare. Whilst we argued that patients treated in the private sector may be more satisfied because providers will focus on care that consumers want, it has also been argued that private patients have more consumerists attitudes because they are paying for their care and, as such, have different (higher) expectations of their care [5, 27]. In other words, private sector consumers may be more at risk of being less satisfied even if the care received is quantifiably comparable to that received by public sector consumers. One large study involving 21 European Union countries reported that those who received care from a private facility were less likely to report high levels of satisfaction [19]. In the US, Medicare beneficiaries have been shown to be more satisfied than persons under age sixty-five who are covered by private insurance [28]. In contrast, a study conducted in Pakistan (a developing country), reported that people treated in private facilities were significantly more satisfied across a range of domains including empathy, technical quality and communication, with the exception of ‘time spent with doctors’ [29]. A study conducted in Ethiopia–another developing country—also observed satisfaction to be higher in the private sector [30]. In the US, privately insured patients have also been shown to be more satisfied following surgical consultations in outpatient clinics [31]. A UK study concluded that whether satisfaction is rated more highly amongst patients treated in the private sector compared to the NHS depended on the therapy being evaluated [27]. Patients rated physiotherapy more highly if provided by the private sector, but osteopathy was appraised similarly regardless of sector. The satisfaction levels of parents of publically insured and uninsured children have been observed to be lower than privately insured families [32]. Privately insured HIV patients have been observed to be more satisfied with their relations with their doctors but not their fees [33]. Private patients having cataract surgery are more satisfied that public patients [34]. In the aforementioned European study, patients who received inpatient care (versus outpatient care), or were surgical versus medical patients, independent of sector, were more likely to be satisfied with care [19]. It would appear then that the extent ‘context’ (healthcare sector) or at least individual insurance status, affects consumer satisfaction, is not straightforward and other features of the care provided—such as the type of intervention (surgical or other, inpatient or outpatient) or the domain of satisfaction under evaluation–influence the relationship observed. That the country the healthcare is provided in also influences the relationship observed suggests to us that we cannot be sure how readily the observations from our current study apply to healthcare sectors elsewhere.

That sector did not emerge here as an important factor with respect to consumer satisfaction following arthroplasty may in part be related to the providers we included. We targeted high volume arthroplasty centres who are, by definition, highly experienced with the procedures. It is possible that high volume centres, regardless of sector, will provide care more in line with consumer expectations. For almost all satisfaction domains monitored here, the overwhelming majority in both sectors were ‘satisfied’ or ‘very satisfied’ and overall satisfaction was very high. These observations would appear to support the notion that the care in specialised centres is in line with consumer expectations and, as such, highlights the need to consider if not control for the level of expertise of the facility or provider when benchmarking satisfaction between providers.

Beyond insurance status and characteristics of the healthcare provider, many studies have shown that outcome of care affects reported satisfaction or likelihood to recommend the care provider. A meta-analysis of 221 studies concluded that outcome of care was an important component of patient satisfaction, though it was less highly regarded than overall quality, humaneness and competence [35]. A recent systematic review covering a wide range of settings and clinical conditions concluded that a positive patient healthcare experience was positively associated with patient safety [36]. A study published since the aforementioned review was undertaken observed a positive association between satisfaction and surgical quality including readmission rates [37]. Investigators in France found that satisfaction with the acute care received after arthroplasty predicted 1-year patient-reported health status [14]. Similarly, investigators in the UK reported that overall satisfaction with the outcome of surgery was strongly predicted by satisfaction with the hospital experience together with the surgery meeting pre-operative expectations and achieving satisfactory pain relief [8]. These same investigators observed that patients who are satisfied with their outcome overall are more likely to promote or recommend their hospital to others [38].

In the current study, the presence of a complication experienced during the acute-care period was a predictor of both overall satisfaction and future recommendations for care. However, the relationship was not straightforward. High levels of satisfaction were still observed amongst those with significant complications and lower levels of satisfaction were observed amongst those without complications. This may explain why others have not seen a relationship between complications and satisfaction with care [39].

Intrinsic consumer characteristics have also been associated with satisfaction with healthcare. Previous studies have reported that those who perceive their health status to be poor are less likely to be satisfied than those who report better health [19,40,41]. Consistent with this, we observed that better baseline Oxford score predicted higher overall satisfaction. Older age [18,19,41] and lower levels of education [18,40] have been shown to be associated with higher levels of satisfaction with healthcare. Some have argued these variables are proxies for expectation [19]. Here, neither age or education level were significant predictors of our primary outcomes.

### Strengths and limitations

Our study has several strengths. Our large cohort was mostly from a random selection of hospitals across a range of socio-geographic areas, thus promoting the generalisability of our findings. Whilst the survey we used was developed specifically for this study, it was based on pilot testing together with close reference to the known domains of satisfaction, and adequate reproducibility for components included in the analysis was achieved. Our inclusion of analysis demonstrating that the individual domain questions were significantly related to the global questions provides evidence that the individual care domains we included are in fact related to overall satisfaction and likeliness to recommend to others. We also contend that positive response bias was minimised by conducting surveys using trained personnel not involved with care delivery.

In terms of limitations, as we only focused on high-volume centres, it is unclear whether our results apply to low volume public and private providers. As well, our sample was smaller than originally planned. In theory, the latter undermined our capacity to detect a significant sector effect, but in reality, reference to individual domain responses (Table 6), indicates we detected between-sector differences as small as 8%. Lastly, despite controlling for it indirectly, we did not specifically measure patient expectation. It is possible, therefore, we have not accounted fully for differences in expectation between our public and private sector consumers. Thus, we cannot completely discount the notion that the reason the private sector was not associated with higher satisfaction levels despite presumably having a higher level of investment in satisfaction domains, is that the latter is potentially offset by high expectations amongst its consumers.

## Conclusions

Arthroplasty consumers treated in the private sector do not report higher overall satisfaction with the acute care experience nor are more likely to recommend the hospital provider to others despite reporting higher levels of satisfaction for a minority of specific care domains. The presence of a complication appears to be more important than ‘sector’ in determining these outcomes.

## Supporting Information

S1 Appendix (DOCX)

S2 Appendix (DOCX)
