# Supplementary material for: Is Satisfaction with the Acute-Care Experience Higher amongst Consumers Treated in the Private Sector? A Survey of Public and Private Sector Arthroplasty Recipients
Source: PLoS One. 2016 Aug 4;11(8):e0159799. doi: 10.1371/journal.pone.0159799 (PMC4973896; doi:10.1371/journal.pone.0159799)
Supplement: S1 Appendix — (DOCX) [file pone.0159799.s001.docx]

**S1 APPENDIX**

I’m going to ask you some questions about how satisfied you were with your experience when you were in hospital where you had your surgery. This is about the care you received, not about how satisfied you are with the outcome of your surgery.

1. How satisfied were you with the cleanliness of the facility?

| Very satisfied | Satisfied | Neither satisfied nor dissatisfied | Dissatisfied | Very dissatisfied |
| --- | --- | --- | --- | --- |

c

1. How satisfied were you with the food?

| Very satisfied | Satisfied | Neither satisfied nor dissatisfied | Dissatisfied | Very dissatisfied |
| --- | --- | --- | --- | --- |

1. How satisfied were you with the way the nursing staff communicated with you about your care?

| Very satisfied | Satisfied | Neither satisfied nor dissatisfied | Dissatisfied | Very dissatisfied |
| --- | --- | --- | --- | --- |

c

1. How quickly did the nursing staff usually respond when you needed help?

| Straight away (within 2 min) | Within 2-5 min | More than 5 min | More than 10 min |
| --- | --- | --- | --- |

c

1. How satisfied were you with the therapy provided by the physiotherapists?

| Very satisfied | Satisfied | Neither satisfied nor dissatisfied | Dissatisfied | Very dissatisfied |
| --- | --- | --- | --- | --- |

c

1. How satisfied were you with the number of times you were seen by the physiotherapist

| Very satisfied | Satisfied | Neither satisfied nor dissatisfied | Dissatisfied | Very dissatisfied |
| --- | --- | --- | --- | --- |

1. How many times were you visited by the physio over the course of your acute care stay?____________________________

**8)** How satisfied were you with how well the medical staff (doctors, excluding your surgeon) kept you informed about your recovery?

| Very satisfied | Satisfied | Neither satisfied nor dissatisfied | Dissatisfied | Very dissatisfied |
| --- | --- | --- | --- | --- |

(If the patient did not see any other doctors, then the question is still relevant. The patient may or may not have been satisfied with that)

c**9)** How clearly did the anaesthetist explain your anaesthetic options to you?

| Very clearly | Not very clearly | I could just understand it | I don’t remember |
| --- | --- | --- | --- |

**10)** How clearly did the anaesthetist explain your post-operative pain management?

| Very clearly | Not very clearly | I could just understand it | I don’t remember |
| --- | --- | --- | --- |

C

**11)** How often did the surgeon (the specialist) visit you in hospital? ___________

**12)** How satisfied were you with the number of times the surgeon visited you in hospital?

| Very satisfied | Satisfied | Neither satisfied nor dissatisfied | Dissatisfied | Very dissatisfied |
| --- | --- | --- | --- | --- |

**13)** Would you recommend this hospital to someone who was having the same surgery as you did?

| No, definitely not | I probably would not | I don’t know | I probably would | Yes, definitely |
| --- | --- | --- | --- | --- |

**14)** In general, did you feel there was enough staff working at any point in time to look after everyone safely?

| Yes, always enough | Yes, more often than not | No, never enough | No, often not enough |
| --- | --- | --- | --- |

**15)** Please rate your overall satisfaction with your hospital stay on a scale of 0-100 (0 = not satisfied at all; 100 = extremely satisfied)

**16)** What aspect of care were you most satisfied with? (list 1 only)

**17)** What aspect of care would you most like to see improved? (list 1 only)
