# Supplementary material for: Is Satisfaction with the Acute-Care Experience Higher amongst Consumers Treated in the Private Sector? A Survey of Public and Private Sector Arthroplasty Recipients
Source: PLoS One. 2016 Aug 4;11(8):e0159799. doi: 10.1371/journal.pone.0159799 (PMC4973896; doi:10.1371/journal.pone.0159799)
Supplement: S2 Appendix — (DOCX) [file pone.0159799.s002.docx]

**S2 Appendix**

S2 Table 1. Reproducibility of Likert questions

|  | Unweighted Kappa | Weighted Kappa | Raw agreement |
| --- | --- | --- | --- |
| Q1 - Cleanliness | 0.690 | 0.778 | 93% |
| Q2 - Food | 0.771 | 0.938 | 83% |
| Q3 – Nurse communication | 0.305 | 0.305 | 90% |
| Q4 – Nurse call responsiveness | 0.422 | 0.423 | 71% |
| Q5 – Physiotherapy overall | 0.803 | 0.895 | 90% |
| Q6 – Physiotherapy visits | 0.733 | 0.760 | 85% |
| Q8 – Medical staff communication | 0.468 | 0.756 | 83% |
| Q9 – Anaesthetist: options | 0.697 | 0.601 | 95% |
| Q10 –Anaesthetist: pain relief | 0.695 | 0.707 | 83% |
| Q12 – Surgeon visitation | 0.835 | 0.970 | 95% |
| Q13 – Hospital recommendation | 0.468 | 0.784 | 90% |
| Q14 – Safety and staff numbers | 0.763 | 0.763 | 88% |

S2 Table 2. Reproducibility of continuous scale questions

|  | Week 1 mean (sd) | Week 2 mean (sd) | Mean difference (sd) week-to-week | ICC, 95% CI |
| --- | --- | --- | --- | --- |
| Recalled number of physiotherapy visits overall | 4.8 (2.7) | 4.6 (2.6) | 0.17 (2.6) | 0.53 (0.25 – 0.72) |
| Recalled number of orthopaedic surgeon visits overall | 2.2 (1.3) | 2.3 (1.3) | -0.09 (0.54) | 0.92 (0.85-0.96) |
| Rating of overall satisfaction with the acute care experience. 0-100% | 90.4 (13.7) | 90.9 (9.7) | -0.50 (7.5) | 0.81 (0.66 – 0.89) |

Note: no significant differences between the two assessments for any variable; ICC, intraclass correlation; sd , standard deviation; CI, confidence interval
